# CGPredictor: a systematic integrated analytic tool for mining and examining genome-scale cancer independent prognostic epigenetic marker panels

**DOI:** 10.1186/1752-0509-7-S6-S10

**Published:** 2013-12-13

**Authors:** Wan-Shu Cheng, Jung-Hsien Chiang

**Affiliations:** 1 Department of Computer Science and Information Engineering, National Cheng Kung University, No.1, University Road, Tainan City 70101, Taiwan

**Keywords:** High-Grade Serous Ovarian Cancer, Invasive Breast Carcinoma, DNA methylation, Epigenetic Clustering

## Abstract

**Background:**

Tumor biomarkers are potentially useful in several ways such as the identification of individuals at increased risk of developing cancer, in screening for early malignancies and in aiding cancer diagnoses; tumor biomarkers may also be used for determining prognosis, predicting therapeutic response, patient tracking following curative surgery for cancer and for monitoring therapy. Epigenetic alterations, especially aberrant DNA methylation, are recognized as common molecular alterations in a variety of tumors and also occur during the development of tumors. The Cancer Grade Predictor (CGPredictor) is an extendable package with functions designed to facilitate systematic integrated and rapid analysis of high-throughput methylation through the use of most self-similarity subgroups of patients supported by various validating examinations with regarded to survival outcome to obtain the identity of the target predictor.

**Results:**

We used high-grade serous ovarian cancer (HGSOC) and invasive breast carcinoma (BRCA) to demonstrate the usefulness of the CGPredictor package. The clustering results and the identity predictors worked well and efficiently in producing significant results after various tests were used to validate the usefulness of CGPredictor package. Also, some of the markers for either the HGSOC or BRCA marker panel have been previously reported to reveal significant results. Even when performed using a different platform with an independent large population BRCA dataset for validation, the identity predictor provided an accurate assessment of patient conditions and produced significant results.

**Conclusions:**

CGPredictor package is not a customized analysis tool designed specifically for the identification of only one or a few specific types of cancer but can be applied more broadly; moreover, the results indicate that the extracted predictors may worthy of consideration for further clinical testing to identify their potential usefulness for clinical molecular diagnosis and targeted treatments of patients with HGSOC and BRCA. So, the use of CGPredictor is feasible for examining the statistical significance of specific markers of interest and shows great potential for use with other types of cancers for cancer biomarker mining.

## Background

DNA methylation has attracted a great amount of interest in the field of cancer research and is currently considered to be a common abnormality found during tumor initiation and subsequent cancer progression [1-3]. DNA methylation of CpG islands regulates gene expression patterns in cancers [2,4]. Also, DNA hypermethylation of promoter-associated CpG islands of tumor suppressor, which leads to transcriptional silencing of these genes, has been the most studied epigenetic alteration in human neoplasia [4]. Methylation patterns and gene expression profiles can be measured on a genome-scale with microarrays which enable integration of these data for further identification of genes that are crucial to cancer progression.

An early diagnosis is critical for the successful treatment of many types of cancer. DNA methylation is closely related to the development of cancer [5]. Since DNA methylation occurs early and can be detected in body fluids, it may be of potential use in the early detection of tumors and for determining the prognosis of some patients [1-3]. The potential to use DNA methylation to determine a patient's prognosis, to predict therapeutic response, for surveillance following curative surgery for cancer and to monitor affected critical genes presents researchers with an attractive option for exploring the clinical use of DNA methylation during the treatment of malignancies. A preventive strategy is needed for patients allowing the use of biomarkers designed to guide physicians in the placement of patients into appropriate screening or surveillance programs for the early detection of cancers. Hence, more reliable markers associated with a large population-base of tumors need to be developed for widespread use in the diagnosis and treatment of cancer. The primary goal of CGPredictor package is to identify and examine biomarkers from strong self-similarity pattern on patients' profiles and the package can be paired with various validation methods designed to facilitate the identification of distinct phenotypes in a variety of cancers.

To demonstrate the utility of CGPredictor, we analyzed alterations in DNA methylation in different cancers of 282 patients with HGSOC [6] as well as 241 patients with BRCA [7] using the Cancer Genome Atlas portal. Tables 1 and 2 show the clinical characteristics of the patients considered in this study. We believe CGPredictor allows researchers to use the first systematic approach which can be used to support the mining and examining cancer biomarker candidates followed with various validation analyses and we found it to be highly efficient (see Table 4). Whether performed using HGSOC or BRCA patients, the statistical significance of the predictor and the clustering genes can be examined; also known cancer markers could be identified in the predictors based on previous reports in the literature.

**Table 1:** Characteristics of the HGSOC participants used in the analysis

	O-CIMP-negative	O-CIMP-positive	Total
No. of Patients	81	32	113

Patient Phenotype Age, years			

Median (LQ, UQ)	56(50, 63)	60(56, 70)	57(51, 66)
No. ≤ 40 years old	6	0	6

Survival (in months)			

Median^a ^(LQ, UQ)	20.9(11.7, 34.6)	17.7(6.8, 27.8)	20(10.5, 30.9)

Sex			

Female	81	32	113
Male	0	0	0

LQ, lower quartile; UQ, upper quartile; and CI, confidence interval.

^a^Median survival and corresponding confidence intervals were estimated from the Kaplan-Meier curve

**Table 2:** Characteristics of the BRCA participants used in the analysis

	B-CIMP-negative	B-CIMP-positive	Total
No. of Patients	77	12	89

Patient Phenotype Age, years			

Median (LQ, UQ)	57(46, 66)	66.5(61, 70)	59(47, 68)
No. ≤ 40 years old	8	1	9

Survival (in months)			

Median^a ^(LQ, UQ)	14.3(7.6, 21.1)	14.5(7.9, 18.8)	19.2(10.7, 32.9)

Sex			

Female	77	12	89
Male	0	0	0

LQ, lower quartile; UQ, upper quartile; and CI, confidence interval.

^a^Median survival and corresponding confidence intervals were estimated from the Kaplan-Meier curve

## Methods

The use of CGPredictor requires several major steps. In the clustering step, the function in the CGPredictor package called "kmeans" is used to cluster samples. In the biomarker selection step, the user can set parameters to choose hypermethylation/hypomethylation corresponding to the downregulated/upregulated intensity between the clustered phenotypes. During the predictor performance examination step, the Cox test is calculated with the clustered clinical outcome of distinct phenotypes and the random selection test can be performed for further validation to increase confidence that gene sets have not been selected randomly. Once validated, a bootstrap test was used to examine the significance between the clustering genes and the phenotypes.

First, the beta value matrix is used for the most self-similarity pattern on patients' profiles clustered together by kmeans function in CGPredictor. To extract the biomarker candidates, gene name is used to link the methylation and gene expression matrices. Also, the mean of gene intensity in each cluster group was determined both for gene expression and DNA methylation for subsequent molecular intensity comparison between clustered phenotypes. Then, the filter function in the CGPredictor package can be used to obtain the biomarker candidates which are predictors for corresponding hypermethylation/hypomethylation to downregulated/upregulated genes between phenotypes. Then, the function in CGPredictor for Kaplan-Meier (KM) curves and Cox test with any observed significant differences in survival for different patient groups can be used to estimate the performance of the predictors. To increase the level of statistical confidence and for further validation of the relationship found between clustering genes and the phenotypes and the significance of the predictor, bootstrap and random selection tests can be performed, respectively. The relationship between clustering genes and the distinct subtype of patients could be measured using the bootstrap test. The bootstrap sample datasets are from the original cancer dataset; we used sampling with replacement with a default iteration of 1,000 times. Also, the original clustering genes were used for kmeans clustering in each rebuilt sample set. Then, the sensitivity would be performed for measuring the statistical significance among the 1,000 iteration sampling dataset. Moreover, the random selection test function is designed to randomly select the same number of genes as were originally extracted as biomarker candidates for a specific cancer. The function in CGPredictor can also be used to efficiently test the extracted predictor's significance with the same default of 1,000 iterations (see Table 4). The programing structure in CGPredictor functions is user friendly. It will allow for future procedure extension as long as the development of the new packages follow the recommended input and output methods for data structure of every function of CGPredictor. Also, CGPredictor is highly extendible for user modification with any of the functions which can be implemented by R. CGPredictor is not limited to DNA methylation microarrays and is scalable to various kinds of microarray analysis problems. However, our integrated system is limited to use on MAC and Windows operating systems and cannot be used on Linux systems, for example.

Measuring how confident one can be of the usefulness of the extracted biomarker candidates is very important in cancer biomarker mining. Aside from some basic processing functions in our integrated system, the statistical validation functions play a critical role for examining the extracted biomarker candidates. Users can measure how their confidence in the relationship found between feature and the clustered phenotypes as well as the ability of the predictor to examine the quality and significance of the biomarker candidates they extracted using our package, CGPredictor.

## Results

### Study population

We used the CGPredictor package to analyze 282 HGSOC and 241 BRCA patients using Infinium HumanMethylation27K (Illumina Inc., San Diego, CA, USA) including 27,578 CpG dinucleotides spanning about 14,000 genes accessed from the Cancer Genome Atlas (TCGA) data portal. Furthermore, an analysis of another large independent dataset including 596 BRCA patients was analyzed on a different platform, HumanMethylation450k; this was performed for validation in the proposed R package. In earlier work, the hESC specific gene panel has been found to be enriched in poorly differentiated tumors[8]. Based on the previous reports [8,9], we then compiled related hESC gene sets. ESC over-expressed genes [10], *Nanog*, *Oct4 *and *Sox2 *targets [11], Polycomb targets in hESCs [12], and *Myc *targets [13,14]. Then, the primary analysis was limited to the common gene set including a total of 3,800 genes for subsequent analysis.

### High-grade serous ovarian cancer data analysis and various validations

**Figure 1:**
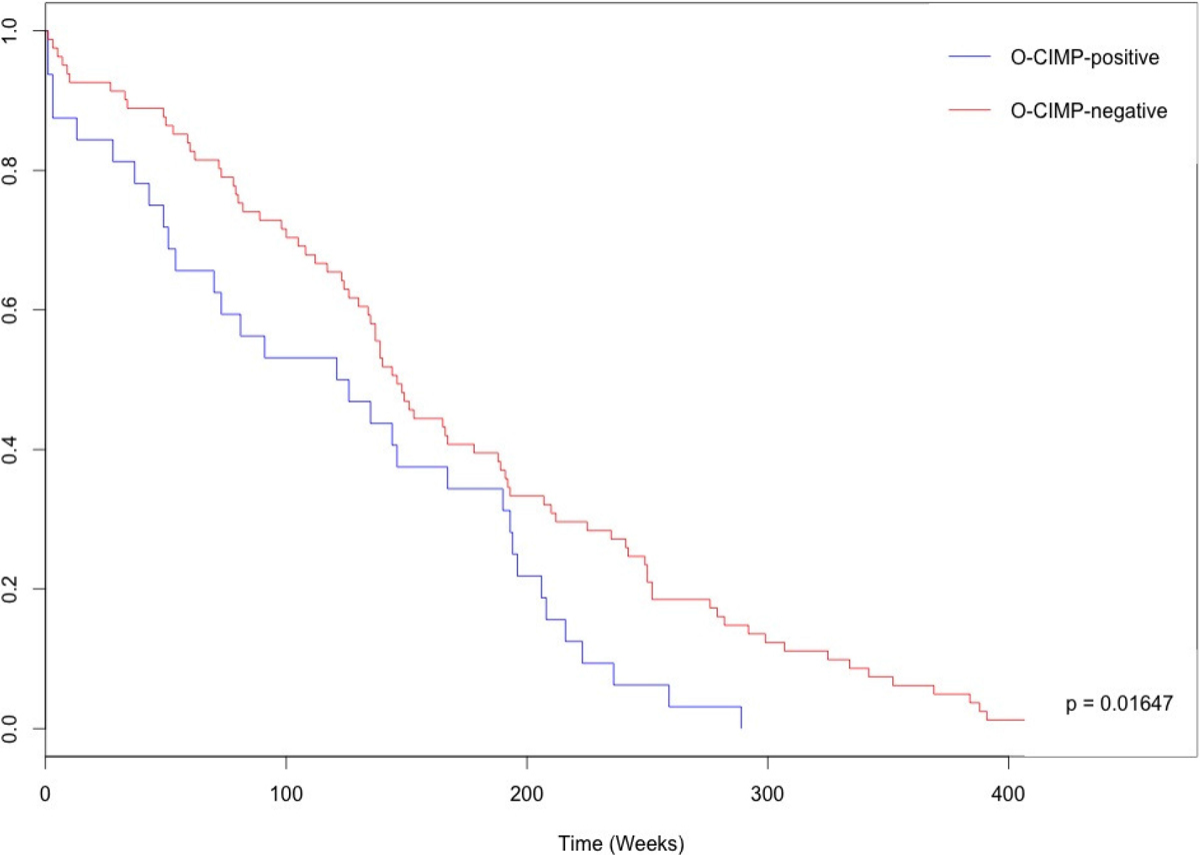
**The relationship between O-CIMP status and patient outcome clustered by the predictor of HGSOC**. O-CIMP-positive (blue lines) and O-CIMP-negative (red) is shown for each KM survival curve. The distinct DNA methylation phenotype within HGSOC patients was identified; a significantly better survival was observed for O-CIMP-negative patients when compared to O-CIMP-positive patients.

After kmeans clustering, the two extreme phenotypes which included the most normal tissues and the most abnormal tissues were labeled as O-CIMP-negative (high grade serous ovarian cancer CpG island methylator phenotype) and O-CIMP-positive, respectively. Toyota, et al. first characterized a CpG island methylator phenotype (CIMP) in human colorectal cancer [15]. When hypermethylated and downregulated genes in HGSOC were retrieved, the 43 extracted genes (as predictor in HGSOC) included *SOX1*, *CALCA*, *DCC*, *GATA4*, and *NID2*, which are the five genes known to be connected to HGSOC. Aside from the five of 43 biomarker candidates which have been reported to have significant usefulness, the KM curve and Cox test for the specific phenotype distinction had a *p*-value of 0.01647 (Figure 1). This indicates the distinct phenotypes clustered by the extracted predictor are significantly different from each other. Furthermore, the predictor for HGSOC were also significant (*p *< 0.0001 after 1,000 iterations) when genes were randomly selected for examining the significance of the extracted predictor. After the bootstrapping with 1,000 iterations, the data was found to be statistically significant (*p *< 0.0001) verified the significance of the clustering results. These results showed that using an extracted predictor from CGPredictor package defined by DNA methylation status is adequate for finding an independent predictor for determining cancer phenotype. Also, the usefulness of the predictor is worth further examination during future clinical testing.

### Breast cancer data analysis and various validations

**Figure 2:**
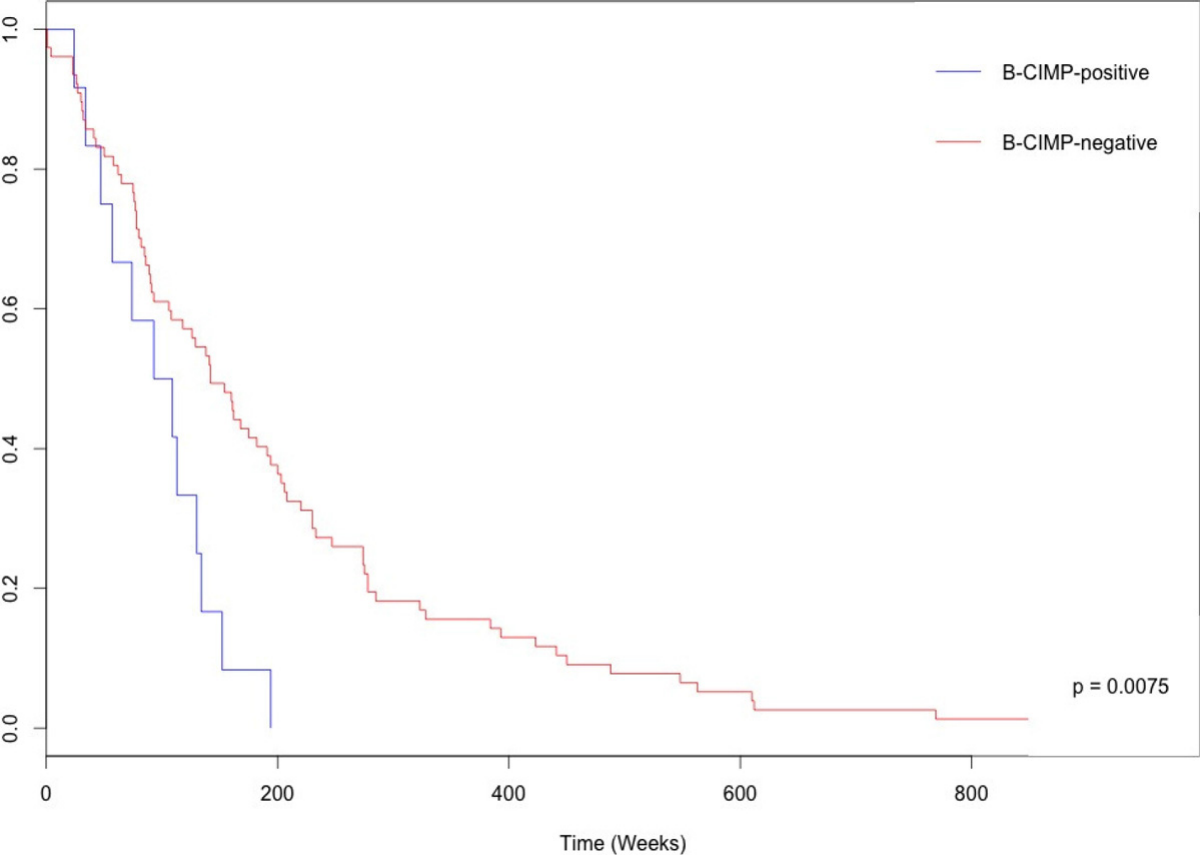
**KM survival curve for the distinct BRCA phenotype**. The significantly better survival for B-CIMP-negative (red) patients compared to B-CIMP-positive (blue) patients was also observed from the plot data; the significant difference between phenotypes was assessed by the predictor evaluated from CGPredictor.

We also considered the 241 BRCA patients which were followed for DNA methylation, mRNA expression and datasets of clinical records as another way of validating the usefulness of CGPredictor. The two distinct phenotypes, B-CIMP-negative (BRCA CpG island methylator phenotype) and B-CIMP-positive were obtained after clustering. After using the same processes as used for HGSOC, ten genes were filtered out as predictors. Among these ten genes, *BMP6* and *GSTP1* have previously been well documented as exhibiting tumor-specific methylation alterations. The two distinct phenotypes were assessed as significant (*p *= 0.0075, Figure 2), after using the function for conducting a Cox test in CGPredictor. The result indicates the gene panel remained a significant predictor of the two distinct phenotypes in patients with BRCA. Furthermore, both the bootstrap test function and the random selection test produced significant results (*p *< 0.0001); the former was implemented in BRCA for examining the relationship between genes for clustering and the distinct phenotypes and the latter test was used for examining the significance of the predicted predictor using randomly selected genes for 1000 repetitions. The result shows the clustering result performed by those clustering genes and the extracted predictor for BRCA were significant.

**Figure 3:**
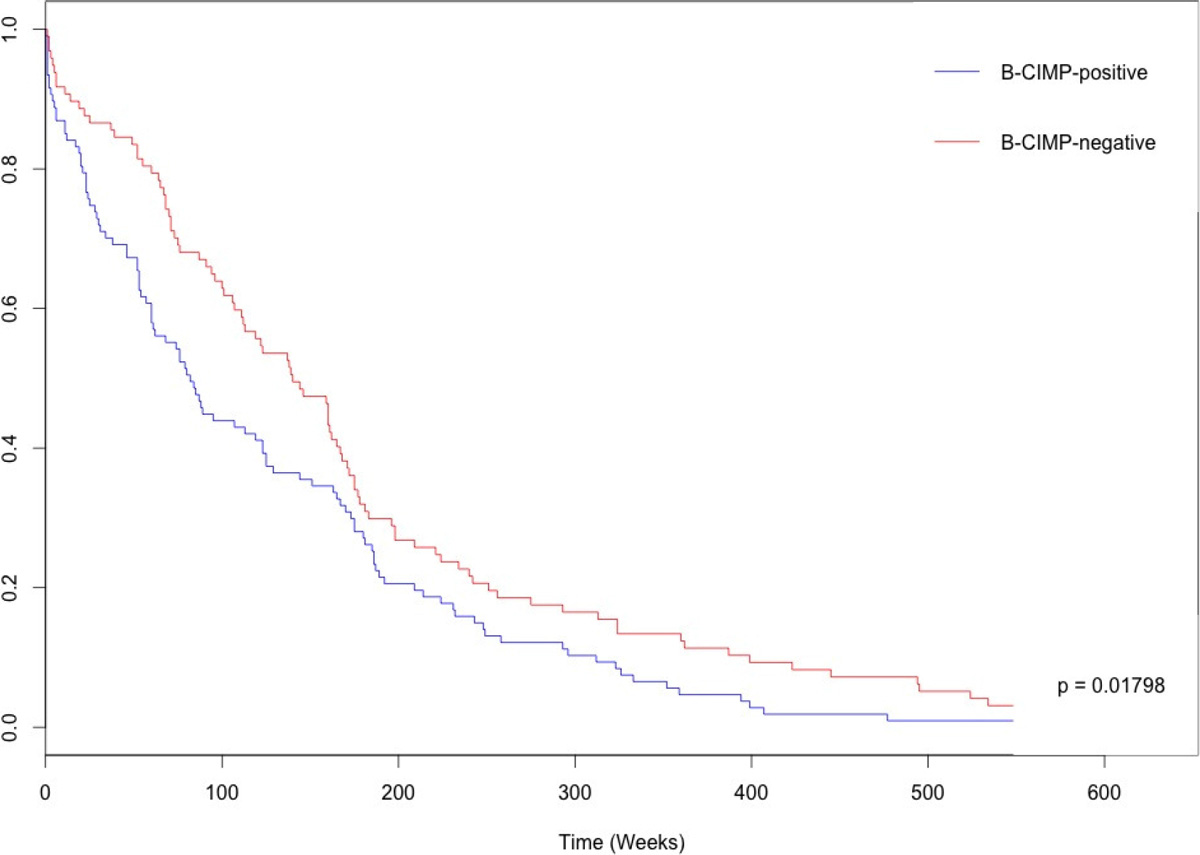
**Kaplan-Meier survival curves comparing B-CIMP-positive (red) and B-CIMP-negative (blue) patients performed with a different independent platform dataset**. Obviously, the significant survival differences were demonstrated for phenotypes by the extracted predictor through the CGPredictor package.

Furthermore, in addition to the support from various validation analysis results and when considering some biomarker candidates which have been significantly reported previously, we used another large independent dataset which was analyzed on a different platform. Specifically, HumanMethylation450k, was performed on 596 BRCA patients in the CGPredictor R package. Table 3 shows the clinical characteristics of those patients. The Cox test supported the use of the identity predictor as a feasible and significant (*p *= 0.01798) predictor which could distinguish the two phenotypes very well for BRCA (Figure 3). The results indicate the devised CGPredictor package, when supported with the various validation methods, could accurately identify a reliable and genome scale cancer independent prognostic epigenetic marker panel. Also, CGPredictor is not simply a tool that custom designed for identifying a specific cancer. CGPredictor can be broadly applied in biomarker mining for various types of cancer.

**Table 3:** Characteristics of the BRCA participants used in the independent validation analysis

	B-CIMP-negative	B-CIMP-positive	Total
No. of Patients	96	108	204

TCGA Patient Phenotype Age, years			

Median (LQ, UQ)	55(45, 66.25)	62(54.7,71)	60(49,68.25)
No. ≤ 40 years old	14	6	20

Survival (in months)			

Median^a ^(LQ, UQ)	35.6(17.5,55.5)	20.3(6.9,46.3)	28.2(12.9,48.3)

Sex			

Female	96	105	201
Male	0	3	3

LQ, lower quartile; UQ, upper quartile; and CI, confidence interval.

^a^Median survival and corresponding confidence intervals were estimated from the Kaplan-Meier curve

**Table 4:** The performance evaluation of the package CGPredictor

	Sample size	Read raw data	Process	Bootstrap (1,000 iterations)	Random selection (1,000 iterations)
**HGSOC**	282 samples 5640 probes	25 sec	8 sec	21 sec	463 sec
**BRCA**	241 samples 3038 probes	16 sec	6 sec	16 sec	393 sec

Intel Core2 2.33 GHz, 2 GB memory, Windows XP

## Discussion

For analysis of the HGSOC and BRCA patient data, CGPredictor package was used to group the most self-similarity pattern on patients' profiles with cancer as subgroups and allowed the identification of 43 and 10 genes as predictors for HGSOC and BRCA, respectively. Significant survival differences were seen in the two distinct phenotypes defined by DNA methylation status (Figure 1 and 2). Previous reports have identified filtered hypermethylation and downregulated genes including *SOX1*, *CALCA*, *DCC*, *NID2*, and *GATA4 *as significant HGSOC markers. As for the predictor for BRCA, *GSTP1 *and *BMP6 *both of these have previously been reported to be significantly related to the presence of BRCA.

Based on these results, to test to see if the relationship between the established clustering gene and the phenotypes was significant, we used bootstrapping with 1,000 iterations; for both HGSOC and BRCA, the clustering results were statistical significance of the clustering result. The identity predictors for each specific type of cancer were examined with the randomly selected genes for the same number of extracted markers in specific cancers for 1,000 iterations. For both the bootstrap test and the random selection test use here, the results were significant (*p *< 0.0001). Moreover, the predictor for BRCA was shown to be capable of indicating significant variations in survival rates using a different independent large population dataset performed using Infinium HumanMethylation450 (Figure 3). These results indicate that the extracted predictor and the clustering results examined from various validations all produce reliable results using CGPredictor; also the CGPredictor package has very good potential for use in mining and examining independent prognostic epigenetic marker panels for other cancers.

When retrieving hypermethylated and downregulated genes indicative of HGSOC, the 43 selected genes includes five which have been previously reported to be connected to HGSOC: *SOX1*, *CALCA*, *DCC*, *GATA4*, and *NID2*. Sox domain proteins are a class of developmentally important transcriptional regulators related to the mammalian testis determining factor SRY [16]. Sox B1 group genes, *Sox1*, *Sox2*, and *Sox3*, are involved in neurogenesis in various species and only the overexpression of *Sox1 *in cultured neural progenitor cells is sufficient to induce neuronal lineage commitment [17]. The methylation of *SOX1 *has been reported as being correlated with the recurrence of ovarian cancer and with overall survival rates for patients with ovarian cancer [18]. As for the gene *GATA4*, it is expressed in most organs and plays a critical role in the development of these organs [19]. *GATA4 *is initially expressed during the formation of extraembryonic endoderm differentiated from the pluripotent embryonic stem cells of the inner cell mass during early embryonic development [20] and is also expressed in human ovarian epithelial cells [21,22]. However, *GATA4 *is often lost in ovarian cancer cells [21,23]. The *GATA4 *gene is believed to dictate distinct pathological pathways leading to serous ovarian carcinomas [24]. Nidogen-2 (*NID2*) is a basement membrane protein. The basement membrane plays an important role in maintaining tissue organization and compartmentalization [25]. Thus, either removal or disruption of the integrity of the basement membrane creates an invasion-permissive environment, often promoting cancer cell proliferation and invasion [26,27]. The loss of nidogen expression has been shown to have a potential pathogenic role in colon and stomach tumorigenesis [28]. Also, the *NID2 *is reported to be a biomarker for ovarian cancer and has been reported to be closely correlated with *CA125 *[29]. *DCC *(Deleted in Colorectal Carcinoma) is an important tumor suppressing gene. *DCC *is a metastasis suppressor gene which targets both proinvasive and survival pathways in a cumulative manner in combination with other genes [30]. Previous report indicated 52% of malignant ovarian cancers did not express the *DCC *gene, and also suggested a significant correlation exists between *DCC *expression and ovarian cancer [31]. As for the promoter of *CALCA*, it was also informative for differentiating methylation between the early stages of ovarian disease and the healthy maintenance of control [32].

In related analysis, two well-known genes are among the ten extracted biomarker candidates which is predictor of BRCA. For instance, *BMP6 *and *GSTP1 *are involved in signal transduction and cell detoxification, respectively. These two genes are two of the top ten hypermethylated genes which have been identified and are used to distinguish between cancerous and normal tissues [33] and different kinds of cohorts have been used for these purposes [34]. Both papers [33,34] suggested the genes might be useful predictors for developing epigenetic-based predictive and prognostic biomarkers for breast cancer. A previous study has also tested from women with palpable lesions suspicious of breast cancer for aberrant promoter hypermethylation, and the *GSPT1 *candidate gene can be easily detected in fine needle aspirated washings. Promoter hypermethylation in benign and malignant lesions was more commonly found in *GSPT1 *than the reported candidate genes [35]. Another previous study determined the frequency of aberrant methylation of *GSTP1 *candidate gene in primary breast cancer tissue for patients with predominantly advanced cancers and suggested that *GSTP1 *is potentially important in the early diagnosis of breast cancer [36].

## Conclusions

The detection of cancer-specific alterations in DNA methylation warrants further investigation because it provides a potential benefit in the early diagnosis of cancer as well as in the evaluation of the prognosis and therapeutic responsiveness of patients. We developed an effective and flexible tool for mining and examining predictors supported by systematic analysis. In addition to efficiently performing the analysis, the CGPredictor package has a variety useful functions which can assist researchers in examining the statistical significance of predictors/specific genes of interest as well as clustering results. With these significant results and based on the fact that some significant genetic markers have been reported previously in the literature for both HGSOC and BRCA, our findings provide further support for idea that CGPredictor package has great potential for mining and examining genome scale independent prognostic epigenetic marker panels for various cancers and also support the potential of the retrieved predictors future clinical testing.

## Availability

CGPredictor R package is implemented in R and is freely available at http://goo.gl/DVqni. A vignette with detailed descriptions of the functions and examples is included.

## Abbreviations

CGPredictor: Cancer Grade Predictor; HGSOC: high-grade serous ovarian cancer; BRCA: breast invasive carcinoma; KM: Kaplan-Meier; TCGA: the Cancer Genome Atlas; O-CIMP: high-grade serous ovarian cancer CpG island methylator phenotype; CIMP: CpG island methylator phenotype; B-GIMP: breast invasive carcinoma CpG island methylator phenotype; NID2: Nidogen-2; DCC: Deleted in Colorectal Carcinoma.

## Competing interests

The authors declare that they have no competing interests.

## Authors' contributions

JH supervised the study, and participated in its design and coordination and helped to draft the manuscript. WS developed the methodology, wrote the software, wrote the manuscript and design the study. Both authors read and approved the final manuscript.

## Declaration

The funding for the paper publication was supported in part by Research Grants from the National Science Council, Taiwan (NSC 102-2221-E-006-236)

This article has been published as part of *BMC Systems Biology *Volume 7 Supplement 6, 2013: Selected articles from the 24th International Conference on Genome Informatics (GIW2013). The full contents of the supplement are available online at http://www.biomedcentral.com/bmcsystbiol/supplements/7/S6.

## References

[B1] Das PM Singal R DNA Methylation and Cancer Journal of Clinical Oncology 2004 7 4632 4642 10.1200/JCO.2004.07.151 15542813

[B2] Kim M Lee J Sidransky D DNA methylation markers in colorectal cancer Cancer and Metastasis Reviews 2010 7 181 206 10.1007/s10555-010-9207-6 20135198

[B3] Heyn H Esteller M DNA methylation profiling in the clinic: applications and challenges Nat Rev Genet 2012 7 679 692 10.1038/nrg3270 22945394

[B4] Esteller M Epigenetics in Cancer New England Journal of Medicine 2008 7 1148 1159 10.1056/NEJMra072067 18337604

[B5] Jones PA Baylin SB The fundamental role of epigenetic events in cancer Nat Rev Genet 2002 7 415 428 12042769 10.1038/nrg816

[B6] Network TCGAR Integrated genomic analyses of ovarian carcinoma Nature 2011 7 609 615 10.1038/nature10166 21720365 PMC3163504

[B7] Network TCGAR Comprehensive molecular portraits of human breast tumours Nature 2012 7 61 70 10.1038/nature11412 23000897 PMC3465532

[B8] Ben-Porath I Thomson MW Carey VJ Ge R Bell GW Regev A Weinberg RA An embryonic stem cell-like gene expression signature in poorly differentiated aggressive human tumors Nat Genet 2008 7 499 507 10.1038/ng.127 18443585 PMC2912221

[B9] Sperger JM Chen X Draper JS Antosiewicz JE Chon CH Jones SB Brooks JD Andrews PW Brown PO Thomson JA Gene expression patterns in human embryonic stem cells and human pluripotent germ cell tumors Proc Natl Acad Sci U S A 2003 7 13350 13355 10.1073/pnas.2235735100 14595015 PMC263817

[B10] Assou S Le Carrour T Tondeur S Ström S Gabelle A Marty S Nadal L Pantesco V Réme T Hugnot JP A Meta-Analysis of Human Embryonic Stem Cells Transcriptome Integrated into a Web-Based Expression Atlas STEM CELLS 2007 7 961 973 10.1634/stemcells.2006-0352 17204602 PMC1906587

[B11] Boyer LA Lee TI Cole MF Johnstone SE Levine SS Zucker JP Guenther MG Kumar RM Murray HL Jenner RG Core Transcriptional Regulatory Circuitry in Human Embryonic Stem Cells Cell 2005 7 947 956 10.1016/j.cell.2005.08.020 16153702 PMC3006442

[B12] Lee TI Jenner RG Boyer LA Guenther MG Levine SS Kumar RM Chevalier B Johnstone SE Cole MF Isono K-i Control of Developmental Regulators by Polycomb in Human Embryonic Stem Cells Cell 2006 7 301 313 10.1016/j.cell.2006.02.043 16630818 PMC3773330

[B13] Figueroa ME Skrabanek L Li Y Jiemjit A Fandy TE Paietta E Fernandez H Tallman MS Greally JM Carraway H MDS and secondary AML display unique patterns and abundance of aberrant DNA methylation Blood 2009 7 3448 3458 10.1182/blood-2009-01-200519 19652201 PMC2765680

[B14] Li Z Van Calcar S Qu C Cavenee WK Zhang MQ Ren B A global transcriptional regulatory role for c-Myc in Burkitt's lymphoma cells Proc Natl Acad Sci U S A 2003 7 8164 8169 10.1073/pnas.1332764100 12808131 PMC166200

[B15] Toyota M Ahuja N Ohe-Toyota M Herman JG Baylin SB Issa J-PJ CpG island methylator phenotype in colorectal cancer Proc Natl Acad Sci U S A 1999 7 8681 8686 10.1073/pnas.96.15.8681 10411935 PMC17576

[B16] Bowles J Schepers G Koopman P Phylogeny of the SOX Family of Developmental Transcription Factors Based on Sequence and Structural Indicators Developmental Biology 2000 7 239 255 10.1006/dbio.2000.9883 11071752

[B17] Kan L Israsena N Zhang Z Hu M Zhao L-R Jalali A Sahni V Kessler JA Sox1 acts through multiple independent pathways to promote neurogenesis Developmental Biology 2004 7 580 594 10.1016/j.ydbio.2004.02.005 15110721

[B18] Su H-Y Lai H-C Lin Y-W Chou Y-C Liu C-Y Yu M-H An epigenetic marker panel for screening and prognostic prediction of ovarian cancer Int J Cancer 2009 7 387 393 10.1002/ijc.23957 18942711

[B19] Kuo CT Morrisey EE Anandappa R Sigrist K Lu MM Parmacek MS Soudais C Leiden JM GATA4 transcription factor is required for ventral morphogenesis and heart tube formation Genes & Development 1997 7 1048 1060 10.1101/gad.11.8.1048 9136932

[B20] Capo-chichi CD Rula ME Smedberg JL Vanderveer L Parmacek MS Morrisey EE Godwin AK Xu X-X Perception of differentiation cues by GATA factors in primitive endoderm lineage determination of mouse embryonic stem cells Developmental Biology 2005 7 574 586 10.1016/j.ydbio.2005.07.037 16162334

[B21] Capo-chichi CD Roland IH Vanderveer L Bao R Yamagata T Hirai H Cohen C Hamilton TC Godwin AK Xu X-X Anomalous Expression of Epithelial Differentiation-determining GATA Factors in Ovarian Tumorigenesis Cancer Research 2003 7 4967 4977 12941822

[B22] Caslini C Capo-chichi CD Roland IH Nicolas E Yeung AT Xu XX Histone modifications silence the GATA transcription factor genes in ovarian cancer Oncogene 2006 7 5446 5461 10.1038/sj.onc.1209533 16607277 PMC7523731

[B23] Wakana K Akiyama Y Aso T Yuasa Y Involvement of GATA-4/-5 transcription factors in ovarian carcinogenesis Cancer Letters 2006 7 281 288 10.1016/j.canlet.2005.10.039 16337738

[B24] Cai KQ Caslini C Capo-chichi CD Slater C Smith ER Wu H Klein-Szanto AJ Godwin AK Xu X-X Loss of GATA4 and GATA6 Expression Specifies Ovarian Cancer Histological Subtypes and Precedes Neoplastic Transformation of Ovarian Surface Epithelia PLoS ONE 2009 7 e6454 10.1371/journal.pone.0006454 19649254 PMC2715102

[B25] Yurchenco PD Amenta PS Patton BL Basement membrane assembly, stability and activities observed through a developmental lens Matrix Biology 2004 7 521 538 10.1016/j.matbio.2003.10.006 14996432

[B26] Sherwood DR FOS-1 promotes basement membrane removal during anchor cell invasion in C. elegans. Cell 2005 7 951 962 10.1016/j.cell.2005.03.031 15960981

[B27] Bassi DE Lopez De Cicco R Cenna J Litwin S Cukierman E Klein-Szanto AJP PACE4 Expression in Mouse Basal Keratinocytes Results in Basement Membrane Disruption and Acceleration of Tumor Progression Cancer Research 2005 7 7310 7319 10.1158/0008-5472.CAN-05-1213 16103082

[B28] Ulazzi L Sabbioni S Miotto E Veronese A Angusti A Gafa R Manfredini S Farinati F Sasaki T Lanza G Negrini M Nidogen 1 and 2 gene promoters are aberrantly methylated in human gastrointestinal cancer Molecular Cancer 2007 7 17 10.1186/1476-4598-6-17 17328794 PMC1831485

[B29] Kuk C Gunawardana CG Soosaipillai A Kobayashi H Li L Zheng Y Diamandis EP Nidogen-2: A new serum biomarker for ovarian cancer Clinical Biochemistry 2010 7 355 361 10.1016/j.clinbiochem.2009.10.012 19883638 PMC3109863

[B30] Rodrigues S De Wever O Bruyneel E Rooney RJ Gespach C Opposing roles of netrin-1 and the dependence receptor DCC in cancer cell invasion, tumor growth and metastasis Oncogene 2007 7 5615 5625 10.1038/sj.onc.1210347 17334389

[B31] Meimei L Peiling L Baoxin L Changmin L Rujin Z Chunjie H Lost expression of DCC gene in ovarian cancer and its inhibition in ovarian cancer cells Medical Oncology 2011 7 282 289 10.1007/s12032-009-9400-z 20054719

[B32] Liggett TE Melnikov A Yi Q Replogle C Hu W Rotmensch J Kamat A Sood AK Levenson V Distinctive DNA methylation patterns of cell-free plasma DNA in women with malignant ovarian tumors Gynecologic Oncology 2011 7 113 120 10.1016/j.ygyno.2010.09.019 21056906 PMC3004216

[B33] Radpour R Kohler C Haghighi MM Fan AXC Holzgreve W Zhong XY Methylation profiles of 22 candidate genes in breast cancer using high-throughput MALDI-TOF mass array Oncogene 2009 7 2969 2978 10.1038/onc.2009.149 19503099

[B34] Radpour R Barekati Z Kohler C Lv Q Bürki N Diesch C Bitzer J Zheng H Schmid S Zhong XY Hypermethylation of Tumor Suppressor Genes Involved in Critical Regulatory Pathways for Developing a Blood-Based Test in Breast Cancer PLoS ONE 2011 7 e16080 10.1371/journal.pone.0016080 21283676 PMC3025923

[B35] Jerónimo C Costa I Martins MC Monteiro P Lisboa S Palmeira C Henrique R Teixeira MR Lopes C Detection of Gene Promoter Hypermethylation in Fine Needle Washings from Breast Lesions Clinical Cancer Research 2003 7 3413 3417 12960130

[B36] Hoque MO Feng Q Toure P Dem A Critchlow CW Hawes SE Wood T Jeronimo C Rosenbaum E Stern J Detection of Aberrant Methylation of Four Genes in Plasma DNA for the Detection of Breast Cancer Journal of Clinical Oncology 2006 7 4262 4269 10.1200/JCO.2005.01.3516 16908936

